# Sampling Strategies in Antimicrobial Resistance Monitoring: Evaluating How Precision and Sensitivity Vary with the Number of Animals Sampled per Farm

**DOI:** 10.1371/journal.pone.0087147

**Published:** 2014-01-23

**Authors:** Takehisa Yamamoto, Yoko Hayama, Arata Hidano, Sota Kobayashi, Norihiko Muroga, Kiyoyasu Ishikawa, Aki Ogura, Toshiyuki Tsutsui

**Affiliations:** 1 Viral Disease and Epidemiology Research Division, National Institute of Animal Health, Kannondai, Tsukuba, Ibaraki, Japan; 2 Animal Products Safety Division, Food Safety and Consumer Affairs Bureau, Ministry of Agriculture, Forestry and Fisheries, Chiyoda-ku, Tokyo Japan; Auburn University, United States of America

## Abstract

Because antimicrobial resistance in food-producing animals is a major public health concern, many countries have implemented antimicrobial monitoring systems at a national level. When designing a sampling scheme for antimicrobial resistance monitoring, it is necessary to consider both cost effectiveness and statistical plausibility. In this study, we examined how sampling scheme precision and sensitivity can vary with the number of animals sampled from each farm, while keeping the overall sample size constant to avoid additional sampling costs. Five sampling strategies were investigated. These employed 1, 2, 3, 4 or 6 animal samples per farm, with a total of 12 animals sampled in each strategy. A total of 1,500 *Escherichia coli* isolates from 300 fattening pigs on 30 farms were tested for resistance against 12 antimicrobials. The performance of each sampling strategy was evaluated by bootstrap resampling from the observational data. In the bootstrapping procedure, farms, animals, and isolates were selected randomly with replacement, and a total of 10,000 replications were conducted. For each antimicrobial, we observed that the standard deviation and 2.5–97.5 percentile interval of resistance prevalence were smallest in the sampling strategy that employed 1 animal per farm. The proportion of bootstrap samples that included at least 1 isolate with resistance was also evaluated as an indicator of the sensitivity of the sampling strategy to previously unidentified antimicrobial resistance. The proportion was greatest with 1 sample per farm and decreased with larger samples per farm. We concluded that when the total number of samples is pre-specified, the most precise and sensitive sampling strategy involves collecting 1 sample per farm.

## Introduction

The emergence of antimicrobial resistance in animals and animal products is a major concern in human health. Consequently, many countries have implemented national monitoring programs for antimicrobial resistance in food production animals or food products. These monitoring programs are aimed at detecting changes in the trends of resistance prevalence and the emergence of resistant microbes [1]. For the same reasons, the Japanese Veterinary Antimicrobial Resistance Monitoring (JVARM) program, a national program for monitoring antimicrobial resistance, has been conducted since 1999 [2] under the direction of the Ministry of Agriculture, Forestry and Fisheries of Japan (MAFF). As a part of monitoring antimicrobial resistance in bacteria obtained from food-producing healthy animals, the prevalence of *Campylobacter spp.*, *Enterococcus spp.*, and *Escherichia coli* is examined by JVARM. JVARM samples a predetermined number of farms in each prefecture every 2 years. In each prefecture, 6 beef cattle farms, 2 pig-fattening farms, 4 layer farms, and 4 broiler farms are sampled. In each selected farm, a sample is taken from an animal and tested for antimicrobial resistance [3].

When designing and planning antimicrobial monitoring systems, the budgetary and human resources required for sampling need to be minimized. The total cost of sampling depends on staff size, their travelling expenditures, and on-site sample collection procedures. It appears that these factors largely depend on the number of farms that are tested, rather than the number of animals sampled per farm. Generally speaking, collecting multiple samples from a single farm is simpler than collecting samples from multiple farms. This provides a motivation for reducing the number of farms that are sampled by JVARM and, in compensation, increasing the number of samples taken from each farm, such that the total sample size remains unchanged.

In many nations such as the Netherlands [4], Denmark [5], Sweden [6], and Canada, the number of samples collected for testing antimicrobial resistance is limited to 1 per farm. The “single sample per farm” strategy is also recommended by the European Food Safety Authority (EFSA), which states that isolates from the same farm are expected to show a similar pattern of resistance; however, the EFSA cites no specific reference for this statement. In addition, although a number of studies have evaluated sampling strategies to understand within-farm prevalence [7], [8], [9], [10], only a few studies have evaluated sampling strategies to assess resistance prevalence at the inter-farm or national levels resistance prevalence.

We sought to provide evidence that would either support this choice of strategy or suggest a better alternative. More specifically, the aim of this study was to determine, on the basis of empirical data, how the number of samples per farm affected the precision and sensitivity of sampling strategies for antimicrobial resistance testing.

## Materials and Methods

### Overview

A total of 1,500 *E. coli* isolates from 30 pig farms in Japan were tested for antimicrobial resistance against 12 drugs. These observational data were used for a simulation study of different sampling strategies. Specifically, we simulated 5 sampling strategies using a bootstrap procedure. Each strategy included a different number of samples per farm, but the same total number of animals sampled (12 animals). The precision of each strategy was evaluated by the deviation of the estimated resistance prevalence. Additionally, the sensitivity of each strategy (the probability of detecting the emergence of antimicrobial resistance that has not been identified previously) was evaluated by the probability that at least 1 resistant isolate was detected.

### Fecal sampling and antibiotic susceptibility testing

Thirty farrow-to-finish pig farms located in 4 prefectures in Japan were selected and sampled between September and October 2011. In each farm, fecal samples were collected from 10 randomly selected fattening pigs aged 150–180 days. Sampling was conducted by field veterinarians with sufficient attention to avoid unnecessary harm to animals. In addition, sampling was conducted under the authorization from farm owners and with the consent of MAFF. Fecal samples were plated on ChromoCult® Coliform Agar (Merck, Darmstadt, Germany), and candidate colonies were confirmed by IMViC (indole, methyl red, Voges-Proskauer, and citrate) reactions. Five *E. coli* isolates were obtained from each fecal sample. All E. coli isolates were tested for antimicrobial resistance using a disc diffusion method [11] with 12 antibiotic disks: oxytetracycline (30 µg; Becton-Dickinson, Franklin Lakes, NJ), sulfisoxazole (250 µg; Becton-Dickinson), sulfamethoxazole plus trimethoprim (23.75 µg plus 1.25 µg, respectively; Becton-Dickinson), ampicillin (10 µg; Nissui Pharmaceutical, Tokyo, Japan), chloramphenicol (30 µg; Nissui), dihydrostreptomycin (10 µg; Nissui), kanamycin (30 µg; Nissui), nalidixic acid (30 µg; Nissui), cefazolin (30 µg; Nissui), gentamicin (10 µg; Nissui), ciprofloxacin (5 µg; Nissui), and cefotaxime (30 µg; Nissui). Antibiotic resistance was determined by the threshold diameter of resistant circles, as described in the instructions attached to the disks. The threshold diameters for each of the antimicrobials were 19, 17, 16, 17, 18, 15, 18, 19, 18, 15, 21 and 23 mm, respectively. The intermediate results were included in the susceptible group, and the results were dichotomized as resistant or susceptible to the different antimicrobials.

### Bootstrap procedure

We compared the precision of different sampling strategies, each of which included a total of 12 animals. We chose to evaluate 12 animals as a total sampling size because the observational data were from 30 source farms of *E. coli* isolates and 12 were available under the single sample per farm strategy with a sufficient number of divisors. Additionally, we set the maximum number of samples per farm at 6 because 10 pigs were sampled per farm in the observational data, and the maximum divisor of 12 is 6. Consequently, 5 strategies were evaluated, sampling 1, 2, 3, 4 or 6 animals per farm, and for all strategies, 12 animals in total were examined (Table 1). Because this study does not focus on the number of isolates per animal, we set the number of isolates to 1 per animal for each strategy.

**Table 1:** List of sampling strategies evaluated by bootstrap resampling.

ID	# Farms	# Animals	# Total
**1**	12	1	12
**2**	6	2	12
**3**	4	3	12
**4**	3	4	12
**5**	2	6	12

To evaluate the precision and sensitivity of sampling strategies, we applied a bootstrap procedure, resampling from the observed data uniformly with replacement [12]. To fully simulate the sampling strategies, bootstrap resampling was performed in 3 steps: at the level of farms, animals, and isolates. In each replication, *n_farm_* farms were selected from 30 farms, and then *n_animal_* animals were selected in each *n_farm_* farm, keeping the total number of selected animals at 12. Finally, 1 isolate was selected within each animal, because the number of isolates from an animal was set to be 1, regardless of the strategy. As a result, 12 samples were selected in each bootstrap replication and this procedure was repeated 10,000 times.

The resistance prevalence—the number of resistant isolates divided by the total number of samples (12)—was calculated for each bootstrap sample for each antimicrobial. The median, 2.5th percentile, 97.5th percentile, and standard deviation (SD) of resistance prevalence obtained from 10,000 bootstrap samples were then calculated to evaluate the precision of each sampling strategy. As an indicator of sampling sensitivity, we calculated the proportion of all bootstrap samples (10,000) that included at least 1 resistant isolate. This calculation was done for each antimicrobial drug separately.

All simulations and statistical analysis were performed using R, version 3.0.0. [13].

## Results

Numbers within parenthesis in the top row of Table 2 present the proportions of *E. coli* isolates resistant to each antimicrobial in the observed data. Resistance prevalence ranged widely—from 0.76 for oxytetracycline to 0.005 for cefotaxime.

**Table 2:** Proportions of *E. coli* isolates resistant to 12 different antimicrobials obtained with 6 different sampling strategies and 10,000 bootstrap replications per strategy.

	**OTC (P = 0.761)**				**SIX (P = 0.635)**				**ST (P = 0.474)**			
**ID**	Med	2.5th	97.5th	SD	Med	2.5th	97.5th	SD	Med	2.5th	97.5th	SD
**1**	0.750	0.500	1.000	0.116	0.667	0.417	0.833	0.130	0.500	0.250	0.750	0.131
**2**	0.750	0.500	1.000	0.129	0.667	0.333	0.917	0.148	0.500	0.167	0.750	0.160
**3**	0.750	0.500	1.000	0.141	0.667	0.333	0.917	0.164	0.500	0.167	0.833	0.181
**4**	0.750	0.417	1.000	0.153	0.667	0.250	0.917	0.179	0.500	0.083	0.917	0.205
**5**	0.750	0.417	1.000	0.172	0.667	0.167	1.000	0.206	0.500	0.000	0.917	0.240
	**ABPC (P = 0.425)**				**CP (P = 0.410)**				**SM (P = 0.390)**			
**ID**	Med	2.5th	97.5th	SD	Med	2.5th	97.5th	SD	Med	2.5th	97.5th	SD
**1**	0.417	0.167	0.667	0.135	0.417	0.167	0.667	0.132	0.417	0.167	0.667	0.132
**2**	0.417	0.167	0.750	0.152	0.417	0.083	0.750	0.154	0.417	0.083	0.667	0.149
**3**	0.417	0.083	0.750	0.167	0.417	0.083	0.750	0.172	0.417	0.083	0.750	0.162
**4**	0.417	0.083	0.750	0.179	0.417	0.083	0.833	0.191	0.417	0.083	0.750	0.176
**5**	0.417	0.083	0.833	0.205	0.417	0.000	0.833	0.221	0.417	0.000	0.750	0.198
	**KM (P = 0.133)**				**NA (P = 0.057)**				**CEZ (P = 0.017)**			
**ID**	Med	2.5th	97.5th	SD	Med	2.5th	97.5th	SD	Med	2.5th	97.5th	SD
**1**	0.083	0.000	0.333	0.094	0.083	0.000	0.250	0.067	0.000	0.000	0.083	0.037
**2**	0.083	0.000	0.333	0.102	0.083	0.000	0.250	0.069	0.000	0.000	0.083	0.037
**3**	0.083	0.000	0.335	0.107	0.000	0.000	0.250	0.068	0.000	0.000	0.083	0.038
**4**	0.083	0.000	0.417	0.113	0.000	0.000	0.250	0.071	0.000	0.000	0.083	0.039
**5**	0.083	0.000	0.417	0.122	0.000	0.000	0.250	0.073	0.000	0.000	0.083	0.039
	**GM (P = 0.013)**				**CPFX (P = 0.009)**				**CTX (P = 0.007)**			
**ID**	Med	2.5th	97.5th	SD	Med	2.5th	97.5th	SD	Med	2.5th	97.5th	SD
**1**	0.000	0.000	0.083	0.032	0.000	0.000	0.083	0.027	0.000	0.000	0.083	0.023
**2**	0.000	0.000	0.083	0.033	0.000	0.000	0.083	0.028	0.000	0.000	0.083	0.024
**3**	0.000	0.000	0.083	0.034	0.000	0.000	0.083	0.029	0.000	0.000	0.083	0.024
**4**	0.000	0.000	0.083	0.035	0.000	0.000	0.083	0.032	0.000	0.000	0.083	0.027
**5**	0.000	0.000	0.083	0.035	0.000	0.000	0.083	0.033	0.000	0.000	0.083	0.027

P = antimicrobial prevalence in 1,500 fecal samples.

ID = identification of sampling strategy shown in Table 1.

OTC = oxytetracycline, SIX = Sulfisoxazole, ST = sulfamethoxazole/trimethoprim, ABPC = ampicillin, CP = chloramphenicol, SM = dihydrostreptomycin, KM = kanamycin, NA = nalidixic acid, CEZ = cefazolin, GM = gentamicin, CPFX = ciprofloxacin, CTX = cefotaxime.

Table 2 presents medians, 2.5th percentiles, 97.5th percentiles, and SDs of prevalence of *E. coli* isolates resistant to each antimicrobial obtained, for each of the 5 sampling strategies. The median values were constant between all sampling strategies and are almost the same as the overall resistance prevalence shown in the top row of Table 2. The 2.5–97.5 percentile interval and the SD were smallest in the sampling strategy that employed 1 animal per farm (ID = 1) in all antimicrobial resistance tests. For most antimicrobials, both the 2.5–97.5 percentile interval and the SD increased with the number of animals sampled per farm. However, no trend of SDs could be reliably detected for antimicrobials that had a low prevalence, such as cefazolin, gentamicin, ciprofloxacin, and cefotaxime.

Table 3 presents the proportions of bootstrap samples detecting 1 or more *E. coli* isolates by antimicrobial drug and sampling strategy. These proportions were greatest in the single animal per farm sampling strategy. For most antimicrobials, the proportion decreased as the number of animals per farm increased. However, for antimicrobials that had high prevalence of resistance, the bootstrap estimate of the proportion always took the value 1.0, irrespective of the sampling strategy, indicating that at least 1 resistant isolate was obtained in all bootstrap samples.

**Table 3:** Proportion of bootstrap samples detecting one or more *E. coli* isolates resistant to 12 different antimicrobials, obtained with 6 different sampling strategies and 10,000 bootstrap replications per strategy.

ID	OTC	SIX	ST	ABPC	CP	SM	KM	NA	CEZ	GM	CPFX	CTX
**1**	1.000	1.000	1.000	0.999	0.999	0.998	0.827	0.513	0.190	0.150	0.109	0.080
**2**	1.000	1.000	0.999	0.998	0.996	0.996	0.806	0.513	0.183	0.148	0.102	0.076
**3**	1.000	1.000	0.996	0.994	0.991	0.992	0.791	0.498	0.174	0.140	0.098	0.069
**4**	1.000	1.000	0.989	0.993	0.984	0.988	0.780	0.494	0.175	0.146	0.095	0.070
**5**	1.000	0.997	0.969	0.981	0.959	0.974	0.739	0.474	0.169	0.133	0.082	0.059

ID = identification of sampling strategy shown in Table 1.

OTC = oxytetracycline, SIX = Sulfisoxazole, ST = sulfamethoxazole/trimethoprim, ABPC = ampicillin, CP = chloramphenicol, SM = dihydrostreptomycin, KM = kanamycin, NA = nalidixic acid, CEZ = cefazolin, GM = gentamicin, CPFX = ciprofloxacin, CTX = cefotaxime.

## Discussion

One of the major objectives of national antimicrobial resistance monitoring is to describe trends of resistance prevalence in the target animal population [14], [15]. Therefore, this study aimed to evaluate the precision of 5 different sampling strategies based on the deviance of estimated prevalence, as assessed by 2.5th–97.5th percentile intervals and SDs. Statistically speaking, a narrow interval and small SD both suggest that the estimated prevalence is more stable and that similar values will be observed across repeated sampling if the target population is static. Thus, the sampling strategy of 1 sample per farm is the most reliable strategy, offering maximum precision. Precision decreased with the increase in the number of samples taken per farm. In the present study, the data used in our bootstrap procedure were obtained from a sampling scheme that was not entirely random; therefore, the true antimicrobial prevalence in the national population remained unknown. Therefore, precise evaluation of the accuracy of these strategies is difficult. Despite this limitation, the agreement of median prevalence between the 5 strategies may indicate a favorable accuracy.

Another objective of national monitoring is to detect an emergence of antimicrobial resistance that was not previously observed in the population [14], [15]. To evaluate the sensitivity of several sampling strategies, we calculated the proportion of bootstrap samples in which 1 or more resistant isolates were detected. As in our evaluation of precision, the sampling strategy with 1 sample per farm exhibited the best sensitivity, and sensitivity decreased with an increase in the number of samples taken per farm. To our knowledge, this is the only study that evaluates the sensitivity of sampling strategies for antimicrobial resistance monitoring based on empirical data. Our comparison of sampling strategy effectiveness by 2 different indicators—precision and sensitivity—will help convince decision makers and stakeholders of our principal result—the superiority of sampling one animal per farm in monitoring programs.

Our result supports the “single sample per farm” strategy that is currently applied in many countries [4], [5], [6] and recommended by the EFSA. As demonstrated by previous studies targeting farm-level monitoring, the best strategies of resistance prevalence monitoring involve taking 1 sample per animal [16], [8]. As an evaluation of national monitoring, our results agree with those of Regula et al. [17], who also demonstrated the advantage of a “single sample per farm” strategy using a bootstrap sampling procedure. Regula et al. obtained bootstrap samples from an artificial dataset of animal and farm bacterial resistance, which was generated from measurements of resistance prevalence. In contrast, our study employs actual field data of 1,500 *E. coli* isolates. Therefore, we believe that our results reflect the true deviance of resistance at the level of farms, animals, and isolates.

This study is subject to some limitations, and therefore, some caution is warranted when interpreting its results. Farms were not selected at random and the number of sampled animals was limited. Additionally, we did not examine animals other than pigs, nor bacteria other than *E. coli*. Although these factors could affect the generalizability of our results, we believe that these limitations are mitigated by our evaluation of sampling strategies using resistance data for a variety of antimicrobials. Furthermore, the actual cost of sampling should also be considered during discussions of the future sampling strategy for the national monitoring system. Future research could examine the cost-effectiveness of different monitoring program designs and the applicability of our results to different livestock and bacteria.

When designing the sampling strategy for a national monitoring system, researchers should specify the minimum change in resistance prevalence that can be detected by the system, as well as the system's threshold for discovery of newly emergent resistance [14]. When a specific sampling strategy needs to be selected with a fixed total number of samples, the single sample per farm strategy should be recommended.
